# First-Principles Insights into the Structural, Electronic, Optical, and Thermoelectric Properties of Novel Halide Double Perovskites Rb_2_InCuX_6_ (X = F, Cl, Br)

**DOI:** 10.3390/nano16100610

**Published:** 2026-05-16

**Authors:** Nabeel Israr, Peichao Zhu, Fawad Ali, Zubair Maroof, Shuaiqi He, Puyang Wu, Haoyang Lu, Weijia Sun, Zhaoxin Wu, Fang Yuan

**Affiliations:** 1 Key Laboratory for Physical Electronics and Devices of the Ministry of Education & Shaanxi Key Lab of Information Photonic Technique, School of Electronic Science and Engineering, Xi’an Jiaotong University, Xi’an 710049, China; nabeelisrar@stu.xjtu.edu.cn (N.I.); zpc360360@stu.xjtu.edu.cn (P.Z.); fawad_ali@stu.xjtu.edu.cn (F.A.); zubair15@stu.xjtu.edu.cn (Z.M.); shuaiqihe@stu.xjtu.edu.cn (S.H.); 1933574811@stu.xjtu.edu.cn (P.W.); luhaoyang@stumail.nwu.edu.cn (H.L.); 2206113855@stu.xjtu.edu.cn (W.S.); 2 Collaborative Innovation Center of Extreme Optics, Shanxi University, Taiyuan 030006, China

**Keywords:** lead-free halide double perovskites, first-principles calculations, electronic band structure, spin–orbit coupling, optoelectronic materials

## Abstract

Lead-free halide double perovskites have emerged as promising candidates for sustainable optoelectronic and thermoelectric applications due to their tunable band gaps, high stability, and non-toxic nature. In this work, we systematically investigate the structural, electronic, optical, and thermoelectric properties of novel double perovskite compounds Rb_2_InCuX_6_ (X = F, Cl, Br) using density functional theory (DFT) combined with spin–orbit coupling (SOC). The structural stability of these materials is confirmed by evaluating the tolerance factor, octahedral factor, and negative formation energy. Accurate band structures obtained via the modified Becke–Johnson (mBJ) potential and SOC reveal direct band gaps of 1.49 eV, 0.91 eV, and 0.56 eV for Rb_2_InCuX_6_ (X = F, Cl, Br), indicating their suitability for solar cell applications. Optical properties, derived from the dielectric functions calculated within the Kramers–Kronig framework over a photon energy range up to 14 eV, show strong absorption peaks in the ultraviolet region, making these materials attractive for high-frequency optical conversion devices. Furthermore, thermoelectric parameters, including the Seebeck coefficient, electrical conductivity, electronic thermal conductivity, and power factor, are computed using the BoltzTraP code. Notably, the figure of merit (*ZT*) approaches 0.80 for Rb_2_InCuF_6_, close to the ideal value of unity, demonstrating excellent thermoelectric performance over a wide temperature range (200–800 K). Our findings establish Rb_2_InCuX_6_ (X = F, Cl, Br) as promising lead-free double perovskites for integrated optoelectronic and thermoelectric applications.

## 1. Introduction

The global demand for energy has grown dramatically over the past few decades. As the foundation of human society, energy is essential not only in quantity but also in quality, driving the need for sustainable and environmentally responsible production [1]. To address the escalating energy crisis, environmentally friendly power sources are urgently required. Silicon-based solar cells have long been considered a direct solution; however, they suffer from challenging manufacturing processes and relatively low power conversion efficiency (PCE), limiting their competitiveness [2]. Consequently, alternative photovoltaic (PV) materials fabricated via simple, cost-effective, and green technologies continue to attract significant scientific interest [3]. The development of highly reliable and efficient PV systems is crucial for harnessing sustainable solar energy [4,5]. Over the past decade, perovskite solar cells (PSCs) have drawn considerable attention due to their remarkable PCE, which rapidly increased from 3.9% to an impressive 27%, along with outstanding bipolar charge mobility, a large absorption coefficient, a tunable band gap, long carrier diffusion lengths, low exciton binding energy, and low trap-state density [6,7]. Despite their broad applicability, lead-based perovskites suffer from intrinsic drawbacks, including poor stability against heat, light, and moisture, as well as the toxicity of lead, all of which hinder their widespread commercial adoption [8,9]. These issues severely restrict their use in PV devices.

Fortunately, halide double perovskites (DPs) with the general formula A_2_M^+^M^+3^X_6_, where two divalent lead ions are replaced by a combination of one monovalent and one trivalent ion, offer a stable and environmentally friendly alternative to lead-based perovskites for various optoelectronic applications. In this formula, A represents a cation, M^+^ and M^3+^ are appropriate monovalent and trivalent metals, respectively, and X denotes a halogen (F, Cl, Br, I). In the crystal lattice, M^+^X_6_ and M^3+^X_6_ octahedra alternate in a corner-sharing arrangement. The thermoelectric, optical, and electrical properties of DPs have sparked research interest across numerous scientifically and technologically important fields, from fundamental materials exploration to practical device applications [10]. DPs can adopt various crystal structures, such as cubic, tetragonal, or orthorhombic, depending on the combination of metal cations and halide anions [11]. The ability to tune their composition and structure enables precise tailoring of their properties. Many DPs possess appropriate band gaps, making them suitable for photodetectors, light-emitting diodes (LEDs), and solar cells [12]. Moreover, DPs have demonstrated efficient light emission, paving the way for their use in LEDs and display technologies [13].

Recently, Cs_2_AgBiBr_6_ has been proposed as a less hazardous alternative to commonly studied lead-based perovskites such as MAPbI_3_ (MA = methylammonium) and CsPbBr_3_ [14,15,16]. Another advantage of DPs over conventional lead halide perovskites is their enhanced stability under ambient conditions and high-intensity light [17]. Several novel double perovskite (DP) materials have been reported, with studies highlighting the optoelectronic and transport properties of compositions where A = Rb, Cs, K; M^+^ = In, Ag, Cu; M^3+^ = Bi, Sb, Ga; and X = I, Br, Cl [18,19,20,21]. For instance, Cs_2_AgBiBr_6_ has been successfully employed in PV and photoelectric devices. Lead-free iron-based DPs such as Cs_2_Ag_x_Na_1−x_FeCl_6_ and Cs_2_CuSbCl_6_ exhibit moderate band gaps of 1.78 eV and 1.66 eV, respectively, making them suitable for solar cells [22,23]. Perovskites based on indium (In) and antimony (Sb) show that the band gaps of Cs_2_AgInCl_6_, Cs_2_AgInBr_6_, and Cs_2_AgSbI_6_ are appropriate for solar cell applications with band gaps 3.02 eV, 1.78 eV, 2.28 eV, respectively [24,25]. Despite these advances, challenges remain, including instability and suboptimal efficiency, which impede practical deployment. When these materials are exposed to moisture, heat, or light, they are more likely to degrade, which over time reduces their performance. Researchers are actively exploring strategies such as interface engineering and encapsulation to enhance DP stability. Furthermore, tuning the optoelectronic properties of halide perovskites is essential for improving device performance, with chemical substitution being the most common and effective approach. The wide compositional tunability of halide perovskites makes it feasible to discover and design novel materials with excellent stability, non-toxicity, and high efficiency for applications in solar panels, thermoelectric generators, and LEDs.

In this context, the present work focuses on Rb_2_InCuX_6_ (X = F, Cl, Br), a class of DP that has received little prior attention. By employing density functional theory (DFT), we aim to elucidate the physical properties of these materials at the atomic level. We systematically investigate the structural, electronic, optical, and thermoelectric characteristics of Rb_2_InCuX_6_ (X = F, Cl, Br) through first-principles calculations, with the goal of identifying promising candidates for perovskite-based optoelectronic devices. Our findings are expected to advance the fundamental understanding of their photoelectric properties, contribute meaningfully to the optoelectronic application industry, and provide valuable guidance for experimental research.

## 2. Computational Methods

Density functional theory (DFT) was employed to investigate the structural properties of the DP compounds Rb_2_InCuX_6_ (X = F, Cl, Br), using the full-potential linearized augmented plane wave (FP-LAPW) method as implemented in the WIEN2k code [26]. Structural optimization, including lattice volume, was performed by fitting the total energy to the Birch–Murnaghan equation of state, in conjunction with the Perdew–Burke–Ernzerhof functional revised for solids (PBEsol-GGA) [27]. During the volume optimization process, a dense k-mesh of dimensions 10 × 10 × 10 was used. The product of the Muffin-Tin radius (*RMT*) and the plane wave cutoff (*K*_max_) was set to 8, and *G*_max_ was defined as 12. In WIEN2K, a cutoff energy of −6.0 Ry is used to designate the energy below which core states are defined. It is expected that core electrons at these energy levels will not mix. For energy and charges, we established convergence conditions of 10^−4^ Ry and 10^−4^ e, respectively.

Although standard DFT has limitations in predicting optoelectronic properties, the Tran-Blaha modified Becke–Johnson (TB-mBJ) potential combined with spin–orbit coupling (SOC) provides accurate band gaps and reliable optoelectronic and other related properties. By successfully reproducing the required band gap values, the TB-mBJ + SOC approach demonstrates excellent adaptability and ease of use [28,29]. The TB-mBJ potential is a semi-local exchange-correlation functional that correctly computes the band gap and is expressed as Equation (1):

(1) $$v_{X,\sigma }^{TB-mBJ}\left(r\right)=cv_{X,\sigma }^{BR}+\left(3c-2\right)\frac{1}{\pi }\sqrt{\frac{5}{6}}\;\sqrt{\frac{t_{\sigma }(r)}{\rho_{\sigma }(r)}}$$

From Equation (1), c is a system-dependent parameter, with *c* = 1 corresponding to the original BJ potential, while $v_{X,\sigma }^{BR}$, is the Becke–Roussel (BR) potential, originally proposed to mimic the slater potential, with the Coulomb potential corresponding to the exact exchange hole [30]. The GGA type’s local potential depends on the electron density ($\rho \;\left(r\right)\;\equiv \sum_{i}^{N}{\left|\psi_{i}\left(r\right)\right|}^{2})$, density gradient (∇ρ (r)), and kinetic-energy density $\left(t\;\left(r\right)\;\equiv \frac{1}{2}\;\sum_{i}^{N}{\left|{\nabla \psi }_{i}\left(r\right)\right|}^{2}\right)$ and can reproduce the OEP exact exchange potential of atoms very well. Thermoelectric properties were calculated using the BoltzTraP2 code, which solves the linearized Boltzmann transport equation (BTE) [31].

## 3. Results and Discussion

### 3.1. Structural Properties: Optimized Structure, Tolerance, Octahedral Factor and Formation Energy

**Figure 1:**
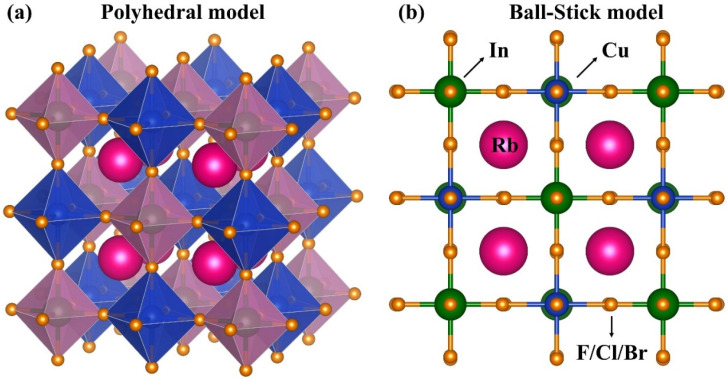
Cubic crystal structure of DPs Rb_2_InCuX_6_ (X = F, Cl, Br) visualized using VESTA: (**a**) polyhedral representation showing In/Cu-centered octahedra; (**b**) ball-and-stick model.

DPs follow the general chemical formula A_2_BB′X_6_, where BX_6_ octahedra occupy positions in the space group Fm$\bar{3}$m [32]. In this structure, the A-site atoms are located at the 8c Wyckoff positions with an oxidation state of +1, while the B-site atoms occupy the 4a sites with an oxidation state of +3. The halide ions (X) reside at the 24e Wyckoff positions with an oxidation state of −1, and the B′-atoms occupy the 4b positions with an oxidation state of +1. Here, A and B′ are monovalent cations, whereas B is trivalent. The title compounds, Rb_2_InCuX_6_ (X = F, Cl, Br), adopt a cubic structure. The atomic positions within the unit cell are Rb at (0.25, 0.25, 0.25), In at (0, 0, 0), Cu at (0.5, 0, 0), and the halogen atoms X at (0.0, 0.0, 0.244) for F, (0.0, 0.0, 0.255) for Cl, and (0.0, 0.0, 0.254) for Br. The variation of halogen position parameter Z from 0.244 in F-based DPs to 0.255 in Br-based DPs highlights a fundamental shift in the relative effective ionic radii of Cu^+^ and In^3+^. While the Shannon radii suggest In^3+^ (0.80 Å) and Cu^+^ (0.77 Å) are much closer, the structural inversion at z = 0.25 suggests that covalence effects dominate in the Br/Cl analogs. The increased hybridization of In-5s states within valance shells of heavier halogens effectively modifies the bond lengths, a phenomenon also observed in related Cs_2_AgInX_6_ DPs [33]. The ground-state lattice volume was optimized by calculating the total energy for a series of unit cell volumes. Because the unit cell contains free atomic sites, the ground state for each volume was determined by ensuring that the atomic forces on each atom were less than 1 mRy/a.u. Figure 1 illustrates the cubic unit cell of Rb_2_InCuX_6_, where each In/Cu atom lies at the center of a halogen octahedron.

The three-dimensional structure of DPs is governed by factors such as ionic radii, ion valences, and the nature of the A, M (here B/B′), and X ions. To maintain a stable 3D DP framework, the ionic radii of A (Rb), M (In/Cu), and X (Cl/Br/I) (R_a_, R_m_, R_x_) must satisfy the octahedral factor (*μ*) and Goldschmidt’s tolerance factor (*τ*), calculated as follows using Equations (2)–(4) [34]:

(2) $$\tau =\frac{\left(R_{A}{+R}_{M}\right)}{\surd 2(R_{M+}R_{X)}}$$

For a DP, the effective ionic radius at the M site (*R*_m_) is obtained from the average of the two B-site cations:

(3) $$R_{M}=\frac{R_{M^{\prime }}+R_{M^{\prime \prime }}}{2}$$

In order to estimate the parameters of the structure, the formula to obtain the *μ* is:

(4) $$\mu =\frac{R_{M}}{R_{X}}$$

To maintain a stable 3D DP structure, the tolerance factor *τ* should lie between 0.8 and 1.0, and the octahedral factor *μ* should fall within 0.41–0.90 [35,36,37]. The *τ* value also provides insight into the degree of symmetry: values close to unity indicate a highly symmetric structure. To achieve such high symmetry, one can select A, M, and X ions with appropriate radii. Alternatively, one may first choose the M and X ions and then select an A-site cation with a radius *R*_a_ that yields a *τ* value equal to or nearly equal to 1. Currently, the most common strategy for obtaining lead-free DPs is to replace two Pb^2+^ cations with one M^+^ and one M^3+^ cation in the form A_2_M^+^M^+3^X_6_ or with a single M^4+^ cation in A_2_M^4+^X_6_ [38].

Through the structural optimization of Rb_2_InCuX_6_ (X = F, Cl, Br), we precisely analyzed the relaxed state energy E (in eV) to ascertain the ground state unit cell volume V_0_. This was achieved by employing the relevant energy and volume data within the framework of the Birch–Murnaghan equation of state (Equation (5)) [39].

(5) $$E=E_{0}+\frac{9V_{o}B_{o}}{16}\left[{\left\{{\left(\frac{V_{o}}{V}\right)}^{\frac{2}{3}}-1\right\}}^{3}{B^{\prime }}_{o}+{\left\{{\left(\frac{V_{o}}{V}\right)}^{\frac{2}{3}}-1\right\}}^{2}\left\{6-4{\left(\frac{V_{o}}{V}\right)}^{\frac{2}{3}}\right\}\right]$$

Table 1 presents a comprehensive analysis of the ideal ground-state structural properties, including lattice constants *a*_0_, total energy *E*_0_, unit cell volume *V*_0_, bulk modulus *B*_0_, pressure derivative of the bulk modulus *B*^p^, Goldschmidt tolerance factor *τ*, octahedral factor *μ*, and formation energy *E*_f_. As the halogen changes from F to Cl to Br, the lattice constants, total energy (*E*_0_), and volume (*V*_0_) all increase, whereas the bulk modulus (*B*_0_) decreases (see Table 1). This trend is attributed to the larger ionic radii of Br^−^ and Cl^−^ compared to F^−^, which also suggests that Rb_2_InCuBr_6_ likely has a smaller band gap than Rb_2_InCuF_6_ [40].

**Figure 2:**
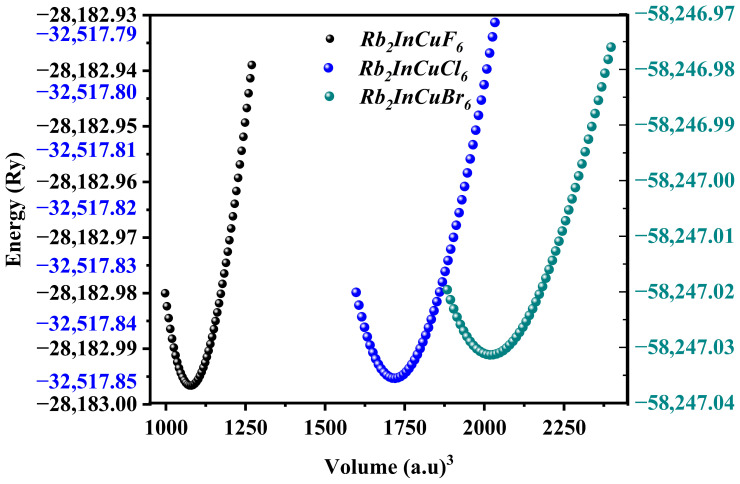
Energy–volume (*E*–*V*) curves for Rb_2_InCuX_6_ (X = F, Cl, Br), obtained from structural optimization using the PBEsol-GGA functional and fitted with the Birch–Murnaghan equation of state. The elevated volume and lower bulk modulus of Rb_2_InCuBr_6_ suggest greater deformation compared to both Rb_2_InCuF_6_ and Rb_2_InCuCl_6_.

Figure 2 displays the energy-volume (*E*-*V*) curves, which clearly show how the total energy varies with volume, confirming that the studied materials are chemically and electronically stable. The negative optimized ground-state energies indicate that Rb_2_InCuX_6_ (X = F, Cl, Br) are stable. To further assess stability, we calculated the formation energy *E*_f_ using Equation (6). The detailed results are summarized in Table 1 [41].

(6) $$E_{f={E_{t}}_{\left({Rb}_{2}CuInX_{6}\right)}-\left(2E_{Rb}-E_{Cu}-E_{In}-6E_{X}\right)bulk}$$

Moreover, the negative formation energies obtained for all three compounds (Table 1) indicate that energy is released during their formation from the constituent elements. This exothermic character confirms that the Rb_2_InCuX_6_ (X = F, Cl, Br) structures are thermodynamically stable under ambient conditions. Furthermore, we utilized Open Quantum Materials Database (OQMD) to accurately predict energy-above -hull values of 0.071, 0.019 and 0.042 eV/atom [42]. While a definitive mapping of the local energy landscape remains a significant computational challenge, the relatively low energy-above-hull values, particularly for the chloride-based phase, suggests that these compounds occupy a sufficiently stable metastable state to permit experimental realization via kinetically controlled synthesis routes [43].

### 3.2. Electronic Properties (Band Structure and Density of States)

**Figure 3:**
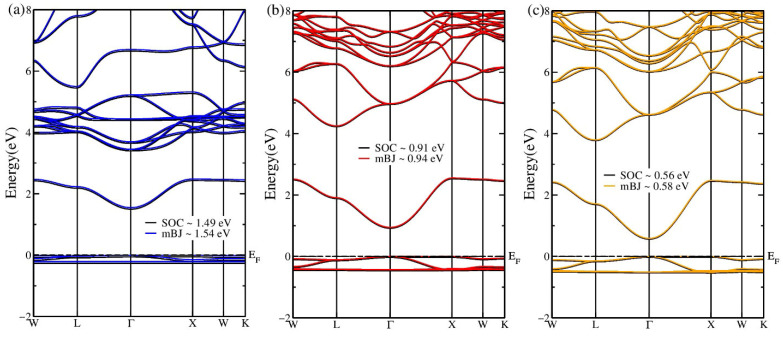
Electronic band structures of (**a**) Rb_2_InCuF_6_, (**b**) Rb_2_InCuCl_6_, and (**c**) Rb_2_InCuBr_6_ calculated using the TB-mBJ potential, including mBJ + SOC. The Fermi level is set to zero. All compounds exhibit a direct band gap at the Γ point.

A thorough understanding of electronic properties is essential for exploring novel applications of materials. In this work, the band structures and density of states (DOS) of Rb_2_InCuX_6_ (X = F, Cl, Br) were systematically investigated using the modified Becke–Johnson (mBJ) potential combined with spin–orbit coupling (SOC), which is known to accurately predict band gaps [44]. The inclusion of SOC ensures more precise and reliable band gap values. As shown in Figure 3, the valence band maximum (VBM) and the conduction band minimum (CBM) are both located at the Γ point for all three compounds, confirming their direct band gap nature. Direct band gap materials are particularly advantageous for optoelectronic applications because they facilitate efficient radiative recombination of charge carriers. Table 2 lists the band gaps obtained from both TB-mBJ and mBJ + SOC calculations. All compounds exhibit semiconducting behavior with the following direct band gaps: 1.54 eV (TBmBJ)/1.49 eV (mBJ + SOC) for Rb_2_InCuF_6_, 0.94 eV/0.91 eV for Rb_2_InCuCl_6_, and 0.58 eV/0.56 eV for Rb_2_InCuBr_6_. The decreasing band gap from F to Br is consistent with the increasing halogen ionic radius. These direct and tunable band gaps make the present compounds promising candidates for various electronic and optoelectronic devices, where the specific band gap value can be tailored for targeted functions [45]. Understanding and manipulating these band gaps is crucial for advancing photovoltaic technologies [46].

**Figure 4:**
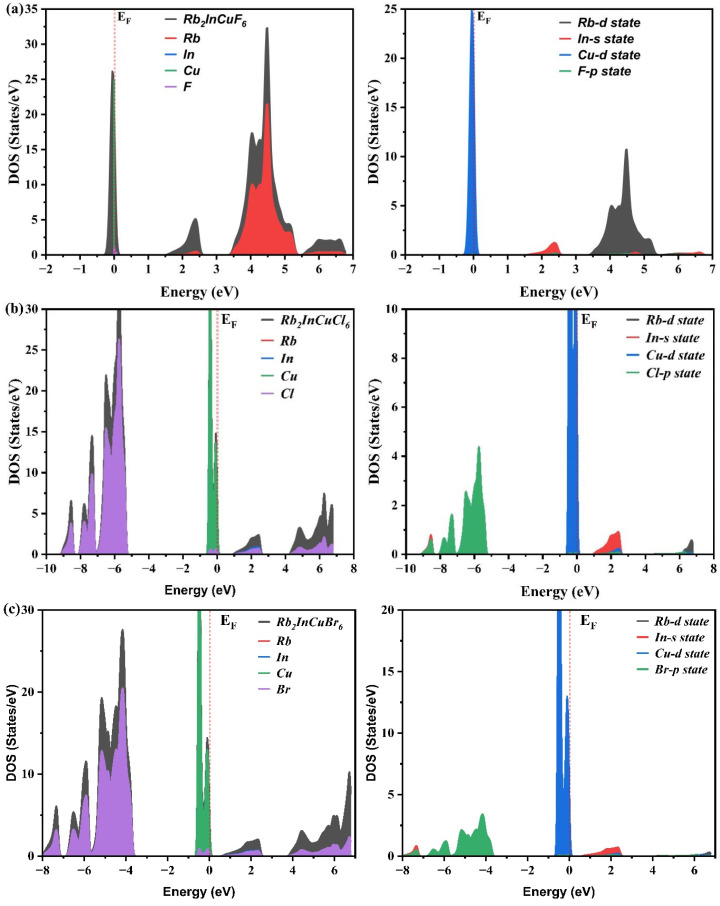
Total and partial density of states (DOS and PDOS) of (**a**) Rb_2_InCuF_6_, (**b**) Rb_2_InCuCl_6_, and (**c**) Rb_2_InCuBr_6_. The Cu-*d* states dominate the valence band near the Fermi level, while In-*s* states contribute significantly to the conduction band. Halide-*p* states appear in the semi-core region, and Rb states contribute deep levels.

The DOS describes the number of electron states available per unit volume per unit energy, while the partial density of states (PDOS) provides detailed insight into orbital hybridization and the origin of electronic transitions. Figure 4a–c presents the calculated DOS and PDOS for Rb_2_InCuX_6_ (X = F, Cl, Br) without spin polarization, revealing several key features. Near the Fermi level, the valence band is predominantly composed of Cu-*d* states hybridized with halide-p states (F-*p*, Cl-*p*, or Br-*p*). This strong hybridization not only determines the valence band maximum (VBM) but also influences the effective mass of holes, which is critical for charge transport. In contrast, the conduction band minimum (CBM) receives dominant contributions from In-*s* states, with minor admixture from Cu-*d* and halide-p states. The spatial separation between Cu-*d* (valence band) and In-*s* (conduction band) character suggests that the primary optical transitions are of the charge-transfer type, i.e., from Cu-*d* to In-*s* orbitals. Meanwhile, Rb states lie deep in energy and do not participate significantly in near-edge hybridization, acting essentially as inert countercations that provide structural stability but do not affect the optoelectronic activity directly. A clear trend is observed upon substituting the halogen from F to Cl to Br. The increasing atomic size and decreasing electronegativity of the halogen lead to a progressive upshift of the halide-p states, which narrows the band gap, consistent with the band structure results in Figure 3 and Table 2. Moreover, the Cu-*d* and halide-*p* hybridization becomes stronger as the halogen becomes more polarizable, enhancing the dispersion of the valence band and potentially reducing the hole effective mass. This is particularly evident for the Br-based compound, where the more diffuse Br-*p* orbitals overlap more effectively with Cu-*d* states, resulting in broader band features and improved carrier mobility. Consequently, the band gap decreases in the order F > Cl > Br, aligning with the trend of the lattice constant and the computed optical absorption edges. In summary, the PDOS analysis not only confirms the direct band gap nature and the origin of electronic transitions but also reveals how halogen substitution systematically modulates the band structure, orbital hybridization, and carrier transport characteristics. These insights are essential for rationally designing Rb_2_InCuX_6_-based perovskites with tailored optoelectronic properties for photovoltaic and light-emitting applications.

To investigate effective mass, we compute the curve of the band edges in the band structure. The curve of the band near the band edge, given by the second derivative of energy with respect to momentum $\frac{d^{2}E}{{dK}^{2}}$, calculates the effective mass. The reciprocal of the curvature yields the effective mass.

(7) $$m^{*}=\frac{\hbar^{2}}{\frac{d^{2}E}{{dK}^{2}}}$$

The investigated effective mass values of electrons and holes are depicted in Table 2. The higher effective mass values of holes reveal flatter bands in the valence band and more curved bands in the conduction band, which is effective for photonic applications. Moreover, the conduction band reveals a higher dispersed nature compared to the valence band, which improves the compound thermoelectric effectiveness. The effective mass values of Rb_2_InCuX_6_ are relatively lower than other DPs, which is consistent with the present work [47,48]. Therefore, improving carrier mobility, a critical feature in the development of advanced electrical and optoelectronic systems, is a benefit of minimal effective mass. Among the calculated values, the Rb_2_InCuBr_6_ compound exhibits lower effective mass, followed by Rb_2_InCuF_6_ and Rb_2_InCuCl_6_. This contributes to greater carrier mobility, making it particularly suitable for optoelectronic applications.

Furthermore, the exciton binding energy ($E_{b}^{ex}$) is investigated based on effective masses of the charge carriers in conjunction with *ε*_1_(0), as listed in Equation (8). The exciton binding energy is the quantity essential to split an exciton that is columbic-bounded into a free charged carrier. Compounds exhibiting the lowest exciton binding energy are preferred for solar cell applications because they provide improved absorbing photons and enhance charge segregation and collection effectiveness.

(8) $$E_{b}^{ex}=\frac{e^{4}}{2{\left(4\pi \varepsilon_{0}\hbar^{2}\right)}^{2}}\frac{\mu_{r}}{\varepsilon_{1}{\left(0\right)}^{2}}\approx 13.56\frac{\mu_{r}}{m_{e}}\frac{1}{\varepsilon_{1}{\left(0\right)}^{2}}$$

where $\mu_{r}$ is the reduced mass is expressed as ($\mu_{r}=\frac{{m_{e}}^{*}{m_{h}}^{*}}{{m_{e}}^{*}{{+m}_{h}}^{*}}$), and $m_{e}$ in term $\frac{\mu_{r}}{m_{e}}$ is the remaining mass of the free electrons (*m*_0_). Rb_2_InCuF_6_ possesses larger effective masses (electron and hole) compared with Rb_2_InCuCl_6_ and Rb_2_InCuBr_6_, indicating lower carrier transport and enhanced dispersion or localization impacts as depicted in Table 2. Furthermore, the predicted $E_{b}^{ex}$ for Rb_2_InCuX_6_ (X = F, Cl, Br) is significantly greater compared to those published for Rb_2_CuAsF_6_ (0.29 eV) and In_2_AgSbCl_6_ (0.058 eV) [49,50]. These elevated values indicate significant Coulomb interactions among holes and electrons, highlighting the potential of Rb_2_InCuX_6_ (X = F, Cl, Br) compounds as superior absorbing material in solar applications.

### 3.3. Optical Properties

To evaluate the potential of Rb_2_InCuX_6_ (X = F, Cl, Br) for solar cell and optoelectronic applications, we performed further calculations using the OPTIC subprogram [46]. A thorough understanding of the frequency-dependent optical constants in response to incident photon radiation is essential for assessing the suitability of any material for such applications. The optical behavior of a material is governed by the complex dielectric function, which describes the electronic response to an electromagnetic field as shown in Equation (9) [51]:

(9) $$\tilde{\varepsilon }\left(\omega \right)=\varepsilon_{1}\left(\omega \right)+i\varepsilon_{2}\left(\omega \right)$$

The real part, *ε*_1_(ω), characterizes the dispersion of light as it interacts with the material surface and can be reliably determined via the Kramers–Kronig dispersion relation [52]. In contrast, the imaginary part, *ε*_2_(ω), represents the material’s light absorption and is accurately computed using momentum matrix elements connecting occupied and unoccupied electronic states. Owing to the cubic symmetry of the present DPs, the dielectric response is isotropic, such that *ε*_xx_ = *ε*_yy_ = ε_zz_ = *ε*. Using *ε*_1_(ω) and *ε*_2_(ω), we subsequently derived several key optical constants, namely, the refractive index *n*(ω), extinction coefficient *k*(ω), optical conductivity *σ*(ω), absorption coefficient *α*(ω), reflectivity *R*(ω), and electron energy loss function *L*(ω) over a photon energy range of 0–14 eV.

**Figure 5:**
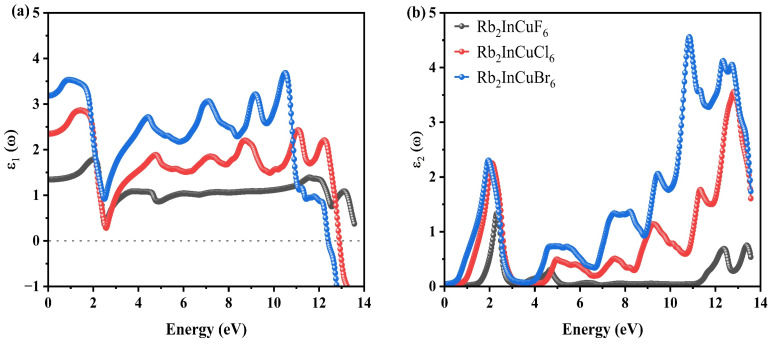

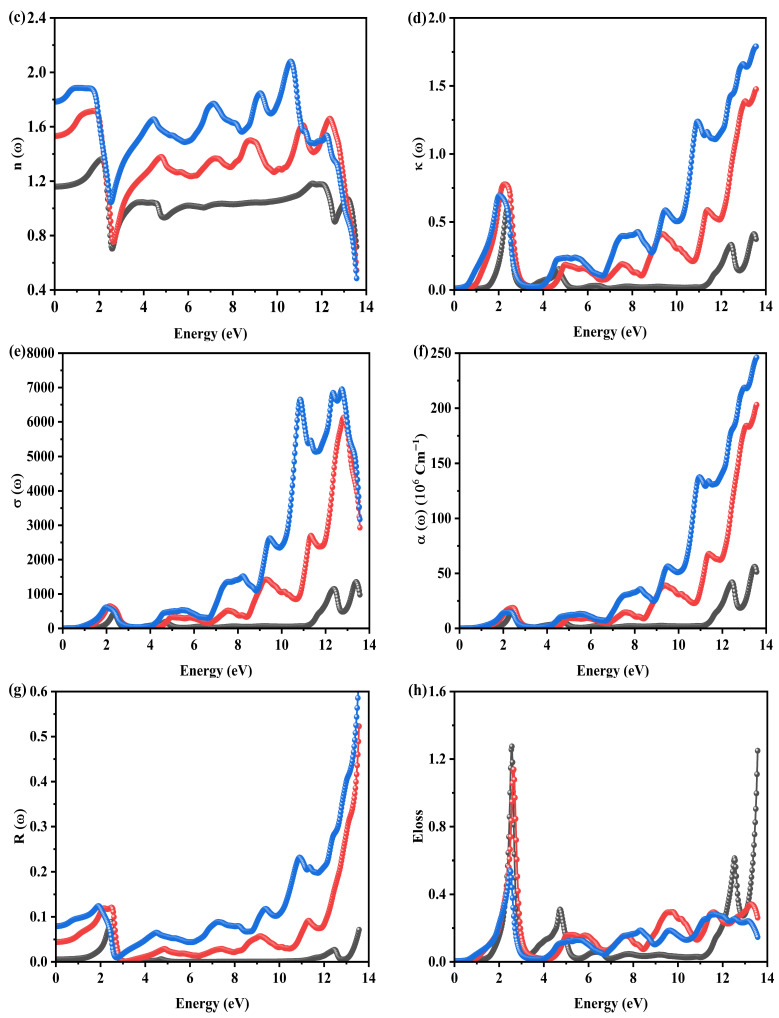
Optical properties of Rb_2_InCuX_6_ (X = F, Cl, Br) as functions of photon energy (0–14 eV): (**a**) real part *ε*_1_(ω), (**b**) imaginary part, *ε*_2_(ω), of dielectric function, (**c**) refractive index *n*(ω), (**d**) extinction coefficient *k*(ω), (**e**) optical conductivity *σ*(ω), (**f**) absorption coefficient *α*(ω), (**g**) reflectivity *R*(ω), and (**h**) electron energy loss function *L*(ω).

Figure 5a reveals a graph depicting the real component of the complex dielectric function, *ε*_1_(ω). A crucial factor, *ε*_1_(0), is a static dielectric constant, which signifies the dielectric property’s value of a material when measured at zero-frequency. The determined values of *ε*_1_(0) for Rb_2_InCuF_6_, Rb_2_InCuCl_6_ and Rb_2_InCuBr_6_ are 1.45, 2.32 and 3.24, respectively. An elevated value of *ε*_1_(0) corresponds to a narrower material bandgap, and conversely, a lower value indicates a wider bandgap. Figure 5a demonstrates that Rb_2_InCuBr_6_ possesses a smaller bandgap compared to Rb_2_InCuF_6_ and Rb_2_InCuCl_6_. The static values of *ε*_1_(ω) and the bandgap (*E*_g_) align with Penn’s model [53], stipulating that *ε*_1_ > 1, as listed in Equation (10):

(10) ε_1_ (0) ≈ 1 + (ℏω_p_/E_g_)

Beginning with the zero-frequency limit, the real part *ε*_1_(ω) of the studied compounds achieved its peak value before commencing a downward trajectory. Notably, within a specific frequency range, the *ε*_1_(ω) value for Rb_2_InCuCl_6_ transitioned to negative, indicating a significant shift in behavior. Therefore, when subjected to light with an energy range of 13.2 eV to 12.8 eV, it exhibits metallic properties instead of dielectric characteristics, rendering it highly suitable for use in radiation protection layers for both Rb_2_InCuCl_6_ and Rb_2_InCuBr_6_, correspondingly. Figure 5a reveals supplementary peaks as the photon energy *E*(eV) escalates. The *ε*_1_(ω) max spectrum peak is detected at 2.31 eV, 1.74 eV and 10.21 eV for Rb_2_InCuF_6_, Rb_2_InCuCl_6_ and Rb_2_InCuBr_6_.

As delineated in Figure 5b, the *ε*_2_(ω) spectra reveal threshold points at 1.64 eV, 0.98 eV and 0.48 eV for Rb_2_InCuF_6_, Rb_2_InCuCl_6_ and Rb_2_InCuBr_6_, which are intimately linked to their respective bandgaps. Moreover, the distinguished peaks represent the pinnacle of scattering that occurs when polarized materials allow light to pass at their resonance frequency. This specific frequency triggers oscillations in atomic dipoles, significantly amplifying dispersion and promoting the seamless transmission of light through surfaces [54]. The imaginary component of dielectric function, *ε*_2_(ω), depicted in Figure 5b, reveals that the peak values for Rb_2_InCuCl_6_ and Rb_2_InCuBr_6_ converge at 2.31 eV. The residual peaks situated within the deep-ultraviolet light spectrum are attributed to electronic transitions from the upper valence bands to the conduction bands, distant from the Fermi surface. This phenomenon suggests promising potential for ultraviolet-light detection and photocatalysis. Additionally, the investigated compounds exhibit a wide absorption spectrum, rendering them highly suitable for a variety of optical applications.

The refractive index of an optical material serves as a vital parameter that significantly impacts its performance and effectiveness in a wide array of sophisticated optical applications. This characteristic is particularly important in the design and implementation of technologies such as photonic crystals, which are engineered to manipulate light at the nanoscale; waveguides, which are essential for guiding light through various media with minimal loss; solar cells, which convert sunlight into electrical energy; and detectors, which are employed to sense and measure light for various purposes. The refractive index determines how light propagates through these materials, affecting their efficiency, sensitivity, and overall functionality. Therefore, understanding and optimizing the refractive index is fundamental for enhancing the capabilities of modern optical devices and systems across numerous fields, including telecommunications, renewable energy, and sensor technology [55,56]. Mathematically, the refractive index can be expressed as:

(11) $$n=\;\frac{1}{\sqrt{2}}[(\varepsilon_{1}+(\varepsilon_{1}^{2}+\varepsilon_{2}^{2}{)}^{\frac{1}{2}}{)]}^{\frac{1}{2}}$$

Figure 5c presents the refractive index *n*(ω) spectra for Rb_2_InCuF_6_, Rb_2_InCuCl_6_ and Rb_2_InCuBr_6_. Notably, the zero-frequency refractive indices, *n*(0), for Rb_2_InCuF_6_, Rb_2_InCuCl_6_ and Rb_2_InCuBr_6_ were ascertained to be 1.2, 1.5 and 1.8, respectively. These measurements are in accordance with the static values of the dielectric function *ε*_1_(0), affirming the relationship between them:

(12) $$n\left(0\right)=\sqrt{\varepsilon_{1}\left(0\right)}$$

Surpassing the zero frequency, the curves reached their zenith for Rb_2_InCuF_6_, Rb_2_InCuCl_6_ and Rb_2_InCuBr_6_, registering peak values of 1.28 at 2.24 eV, 1.70 at 1.92 eV and 2.14 at 10.82 eV, respectively. Upon attaining their peak values, the refractive indices of the compounds commence a decline, eventually falling below specific energy thresholds. The occurrence where the refractive index *n*(ω) exceeds unity signifies that photons experience a reduction in speed due to their interaction with the surface electrons of the material, causing a delay as they pass through these substances. On the other hand, when the *n*(ω), falls below the value of one, it suggests that the group velocity, represented by *V*_g_ and calculated as *c*/*n*, of the incoming electromagnetic radiation exceeds the speed of light in a vacuum, which is denoted by *c*. This intriguing situation leads to the group velocity *V*_g_ entering a negative range, indicating a significant alteration in the characteristics of the medium from a linear regime (where the response of the material is directly proportional to the incident light intensity) to a non-linear regime (where the response becomes more complex and dependent on higher-order effects). This extraordinary phenomenon endows the material with superluminal properties, allowing it to exhibit behaviors that challenge our conventional understanding of causality and the limitations imposed by the speed of light. Such materials, therefore, open up fascinating avenues for exploration in fields such as optics, telecommunications, and theoretical physics, where the implications of surpassing the light speed barrier could lead to groundbreaking advancements in technology and our comprehension of the underlying principles governing wave propagation in various media [57,58].

The extinction coefficient *k*(ω) evaluates the ability of the material to reduce the light intensity. The optical constant *k* can be expressed using the dielectric function, as follows:

(13) $$k\;=\;\frac{1}{\sqrt{2}}[(-\varepsilon_{1}+(\varepsilon_{1}^{2}+\varepsilon_{2}^{2}{)}^{\frac{1}{2}}{)]}^{\frac{1}{2}}$$

The trend of *k*(ω) is similar to that of *ε*_2_(ω), as seen in Figure 5b–d. The *k*(ω) spectrum shows first maxima of 0.56, 0.74 and 0.67 at 1.52 eV, 1.46 eV and 1.32 eV for Rb_2_InCuF_6_, Rb_2_InCuCl_6_ and Rb_2_InCuBr_6_ in the visible region. The *k*(ω) spectrum shows the second-highest peak values of 0.43, 1.48 and 1.81 at 13.5 eV for Rb_2_InCuF_6_, Rb_2_InCuCl_6_ and Rb_2_InCuBr_6_ in the UV region.

A material’s optical conductivity is determined by the amount of free electrons present in it [59]. The optical conductivity, *σ*(ω) (Ω^−1^ m^−1^), increases with a higher imaginary component of the dielectric function *ε*_2_(ω), which corresponds to greater optical absorption. The optical conductivity can be expressed as [60]:

(14) $$\sigma \left(\omega \right)=\frac{\omega }{4\pi }\varepsilon_{2}\left(\omega \right)$$

Figure 5e demonstrates that Rb_2_InCuF_6_, Rb_2_InCuCl_6_ and Rb_2_InCuBr_6_ materials display a broad spectrum of optical conductivity across the spectral range extending from 1.5 to 13.4 eV. This observation indicates that within this specific energy range, the materials are capable of absorbing and responding to light in a significant manner. For Rb_2_InCuF_6_, Rb_2_InCuCl_6_ and Rb_2_InCuBr_6_, the exceptional optical conductivity values of 1405 Ω^−1^ cm^−1^ at 13.4 eV, 6104 Ω^−1^ cm^−1^ at 12.8 eV and 6978 Ω^−1^ cm^−1^ at 12.7 eV, respectively, underscore the potential of these materials for advanced optical applications.

The absorption coefficient is a crucial parameter in understanding the behavior of light as it interacts with different materials. Specifically, it quantifies the extent to which a specific wavelength of light is able to penetrate into a substance before it is absorbed [61]. This coefficient plays a significant role in various fields, including optics, materials science, and engineering, as it helps in predicting how different materials affect the transmission of light. The absorption coefficient can be written as:

(15) $$\alpha \left(\omega \right)=\frac{4\pi }{\lambda }k$$

If a material has a low absorption coefficient, it will not absorb light effectively. The absorption coefficient is significantly affected by two primary factors: the intrinsic properties of the material in question and the specific wavelength of the light being used. Figure 5f illustrates that the *α*(ω) values of the materials under study are minimal within the visible wavelength spectrum. This indicates that the materials exhibit pronounced responsiveness to ultraviolet photons and are well-suited for applications in such conditions. Rb_2_InCuX_6_ (X = F, Cl and Br) material possesses a distinct threshold, beyond which it ceases to absorb light. When photons engage with electrons in their valence states, a photon–electron interaction ensues, leading to highly efficient light absorption once this threshold is surpassed. Notably, the absorption edges exhibit a consistent shift in tandem with the band gaps. The absorption coefficient *α*(ω) exhibits threshold values of 1.92 eV, 1.74 eV and 1.62 eV for Rb_2_InCuF_6_, Rb_2_InCuCl_6_ and Rb_2_InCuBr_6_. Notably, the highest peak values of *α*(ω) reach 58.0 × 10^6^ cm^−1^ at 13.4 eV for Rb_2_InCuF_6_, 183.23 × 10^6^ cm^−1^ at 13.0 eV for Rb_2_InCuCl_6_ and up to 221.9 × 10^6^ cm^−1^ at 13.0 eV for Rb_2_InCuBr_6_. The DPs being studied show a notable enhancement in optical absorption within the Extreme (EUV) range. Detailed analysis of their optical properties confirms that Rb_2_InCuX_6_ (X = F, Cl, Br) is well-suited for optoelectronic uses. Additionally, absorption occurring in the near-infrared spectrum contributes to the reduction in bandgap [62]. The blue shift is observed while replacing the halide atoms from F, Cl and Br, respectively. The elevated peaks are observed for Br-based DPs and then F- and Cl -based DPS, as listed in Figure 5f.

Reflectivity, denoted by *R*(ω), is defined as the amount of energy that propagates back after striking with the surface of the medium. Reflectivity is critical in applications such as solar panels, where materials with low reflectivity are preferred for maximum energy absorption [63]. Mathematically, it can be expressed as follows:

(16) $$R\left(\omega \right)=\frac{(n-1{)}^{2}-k^{2}}{(n+1{)}^{2}-k^{2}}$$

The reflectivity spectra for Rb_2_InCuX_6_ (X = F, Cl, Br) are illustrated in Figure 5g. At the zero-frequency limit, the reflectivity *R*(0) for Rb_2_InCuF_6_ is 0.01, for Rb_2_InCuCl_6_ is 0.068, and for Rb_2_InCuBr_6_ is 0.098. As the photon energy (eV) increases, the reflectivity of these DPs also rises, reaching peak values of 0.084 at 13.6 eV for Rb_2_InCuF_6_, 0.58 at 13.5 eV for Rb_2_InCuCl_6_ and 0.64 at 13.4 eV for Rb_2_InCuBr_6_. Within the designated visible energy range, the reflectivity of Rb_2_InCuX_6_ (X = F, Cl, Br) remains relatively moderate. When it comes to efficiently absorbing light in the UV region, the materials Rb_2_InCuX_6_ (X = F, Cl, Br) exhibit the highest reflectivity peaks. As illustrated in Figure 5f,g, these materials demonstrate remarkably low reflectivity and absorption within the visible energy spectrum. With the background of solar cells, materials characterized by both low reflectivity and low absorption are highly advantageous, as they maximize the capture and conversion of sunlight into electricity. By allowing a greater number of photons to penetrate the cell and minimizing energy losses, these materials substantially enhance overall efficiency. Additionally, these materials prove to be invaluable in applications such as LEDs, display technologies, sensors, photodetectors, and cold light sources [64,65].

The phenomenon known as electron energy loss, denoted as *L*(ω), pertains to the amount of energy that is dissipated when electrons engage in interactions with various materials, which can be expressed as:

(17) $$L\left(\omega \right)=\frac{\varepsilon_{2}\left(\omega \right)}{\varepsilon_{1}^{2}\left(\omega \right)+\varepsilon_{2}^{2}\left(\omega \right)}$$

The electron energy loss spectra for the Rb_2_InCuX_6_ (X = F, Cl, Br) compound, represented by the electron energy loss function *L*(ω), are illustrated in Figure 5h. This figure eloquently delineates the energy dissipation of electrons within the material. The spectral analysis reveals a pronounced loss in the low-energy infrared region, which diminishes as the incident photon energy increases, encompassing the ultraviolet and visible regions. The observed peak at maximum intensity is indicative of plasma resonance, highlighting the plasma frequency. The *L*(ω) spectra unveil distinct peaks indicative of plasma resonance, with peak energies measured at 2.52 eV for Rb_2_InCuF_6_, 2.75 eV for Rb_2_InCuCl_6_ and 2.67 eV for Rb_2_InCuBr_6_. Furthermore, it is evident that energy loss is completely mitigated within the operational regions, underscoring the significance of these materials for optoelectronic applications [66,67,68,69].

### 3.4. Thermal Properties

**Figure 6:**
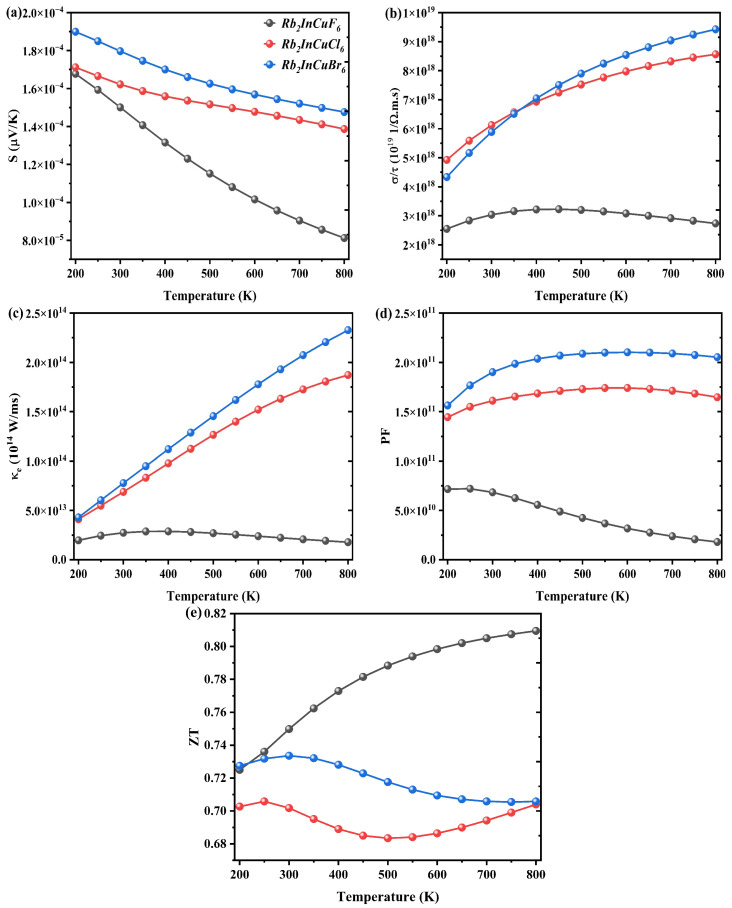
Temperature-dependent thermoelectric properties of Rb_2_InCuX_6_ (X = F, Cl, Br) in the range 200–800 K: (**a**) Seebeck coefficient *S*, (**b**) electrical conductivity divided by relaxation time *σ*/*τ*, (**c**) electronic thermal conductivity divided by relaxation time *κ*_e_/*τ*, (**d**) power factor *P*_F_, (**e**) figure of merit *ZT*. Rb_2_InCuF_6_ shows the highest *ZT* value (0.8 at 800 K), indicating excellent thermoelectric performance.

The thermoelectric properties of Rb_2_InCuX_6_ (X = F, Cl, Br) DPs were evaluated using the BoltzTraP2 code [70]. Electron mobility from the valence band (VB) to the conduction band (CB) occurs upon energy absorption, increasing the majority charge carriers in both n-type and p-type materials. The Seebeck coefficient *S* (µV/K), which quantifies the thermovoltage generated per unit temperature difference [71], is shown in Figure 6a. A larger absolute *S* is desirable for high-performance thermoelectric devices. Rb_2_InCuX_6_ (X = F, Cl, Br) exhibits positive *S* (µV/K) values, indicating p-type conductivity, with holes as the majority carriers. Over the temperature range of 200–800 K, *S* for Rb_2_InCuF_6_ drastically lags, from 1.6 × 10^−4^ to 8.1 × 10^−5^ µV/K, while for Rb_2_InCuCl_6_ and Rb_2_InCuBr_6_, *S* decreases gradually from (1.7/1.3) × 10^−4^ µV/K to (1.8/1.4) × 10^−4^ µV/K. The magnitude and sign of *S* (µV/K) are governed by the electronic band structure and charge-carrier scattering mechanisms.

Figure 6b presents the temperature dependence of electrical conductivity divided by relaxation time (*σ*/*τ*) over the range of 200–800 K. For Rb_2_InCuF_6_, *σ*/*τ* increases non-linearly from 2.5 × 10^18^ to 2.7 × 10^18^ 1/(Ω·m·s) as the temperature rises. In contrast, Rb_2_InCuCl_6_ and Rb_2_InCuBr_6_ exhibit an exponential increase, with values rising from approximately 4.9/4.3 × 10^18^ to 8.5–9.4 × 10^18^ 1/(Ω·m·s). This behavior is consistent with the increase in charge carrier concentration in the conduction band at elevated temperatures. As shown in Figure 6b, Rb_2_InCuBr_6_ consistently exhibits the highest *σ*/*τ* among the three compounds across the entire temperature range, owing to its more dispersive conduction bands (see Section 3.2), which facilitate carrier transport.

Figure 6c displays the temperature-dependent electronic thermal conductivity (*κ*_e_) in units of 10^14^ W/(m·s). A higher *κ*_e_ reflects a greater capacity for heat conduction by electrons. Rb_2_InCuCl_6_ and Rb_2_InCuBr_6_ reveal a pronounced increase in κ_e_ with temperature, reaching (1.8 and 2.3) × 10^14^ W/(m·s) at 800 K, suggesting an enhanced electronic contribution to heat transport, possibly due to modified scattering processes or increased electron mobility. In comparison, Rb_2_InCuF_6_ exhibits lower *κ*_e_ values, with a slow, nearly linear rise. These differences arise from variations in their electronic band structures. The observed increase in *κ*_e_ with temperature is qualitatively consistent with the Wiedemann–Franz law, which states that electronic thermal conductivity is proportional to electrical conductivity, a trend typically seen in metals and degenerate semiconductors [72]. At 800 K, the maximum *κ*_e_ values for Rb_2_InCuCl_6_ and Rb_2_InCuBr_6_ reveal higher conductivity compared with Rb_2_InCuF_6_. The power factor *PF* = *σS*^2^, which reflects the thermoelectric efficiency of a material, is plotted in Figure 6d. Higher *PF* values indicate better thermoelectric performance. Rb_2_InCuF_6_ achieves a peak *PF* of 7.1 × 10^14^ at 200 K, demonstrating lower performance. In contrast, Rb_2_InCuCl_6_ and Rb_2_InCuBr_6_ show higher peak *PF* values of 1.7 × 10^14^ at 500 K and 2.1 × 10^14^ at 600 K, respectively, indicating less efficient thermoelectric energy conversion. Maximizing *PF* is essential for realizing effective heat-to-electricity conversion devices.

Finally, the figure of merit *ZT* is presented as a function of temperature in Figure 6e. Owing to its lower *κ*_e_/*τ* and higher *S*, Rb_2_InCuF_6_ exhibits a *ZT* value that increases steadily across the entire temperature range, reaching 0.80 at 800 K, which is appreciable to the ideal value of 1. This makes Rb_2_InCuF_6_ a highly promising candidate for thermoelectric applications. For Rb_2_InCuCl_6_ and Rb_2_InCuBr_6_, *ZT* reveals some fluctuations but remains relatively stable, with average values of approximately 0.70 at 250 K and 0.73 at 300 K, indicating moderate thermoelectric capability. However, the use of a constant relaxation time approximation may overestimate the thermoelectric performance. The minor variations in *ZT* with temperature reflect subtle changes in material properties that affect thermoelectric efficiency [73]. Overall, efficient thermoelectric material is widely recognized to require a combination of high electrical conductivity, high *S* (µV/K), and low electronic thermal conductivity, which is a set of properties that Rb_2_InCuF_6_ successfully approaches.

## 4. Conclusions

In summary, the structural, electronic, optical, and thermoelectric properties of the lead-free DP Rb_2_InCuX_6_ (X = F, Cl, Br) were systematically investigated using the FP-LAPW method within DFT. The structural stability of these compounds was confirmed by their negative formation energies, as well as by the calculated tolerance and octahedral factors. Electronic band structures, obtained using the mBJ potential including SOC, reveal that all three compounds possess direct band gaps of 1.49 eV (F), 0.91 eV (Cl), and 0.56 eV (Br), making them attractive for photovoltaic and optoelectronic applications. The optical properties, derived from the dielectric function within the Kramers–Kronig framework over a photon energy range of 0–14 eV, exhibit strong absorption in the UV region, underscoring the potential of these materials for high-frequency optical conversion devices. Furthermore, thermoelectric parameters evaluated with the BoltzTraP2 code show that Rb_2_InCuF_6_ achieves a figure of merit (*ZT*) of 0.80 at 800 K, approaching the ideal value of unity and demonstrating excellent thermoelectric performance for power generation and cooling applications. Collectively, these findings establish Rb_2_InCuX_6_ (X = F, Cl, Br) as promising lead-free DPs, offering a viable pathway toward the rational design of high-efficiency, environmentally friendly optoelectronic and thermoelectric devices.

## Figures and Tables

**Table 1:** Calculated lattice parameters, ground state energy *E*_0_ (eV), unit cell volume *V*_0_ (a.u.)^3^, bulk modulus *B*_0_ (GPa), pressure derivative of bulk modulus *B*^p^, Goldschmidt tolerance factor *τ*, octahedral factor *μ*, and formation energy *E*_f_ (eV/atom) for Rb_2_InCuX_6_ (X = F, Cl, Br).

Compounds	$a_{0}\;(Å)$	E_0_ (eV)	V_0_ (a.u)^3^	B_0_ (GPa)	B^p^	τ	µ	E_f_ (eV/Atom)
**Rb_2_In** **Cu** **F_6_**	8.61	−388,005.28	1077.38	69.81	4.92	0.98	0.47	−2.38
**Rb_2_In Cu Cl_6_**	10.06	−442,428.24	1749.82	38.54	4.85	0.97	0.44	−2.35
**Rb_2_In Cu Br_6_**	10.62	−792,492.12	2086.28	31.12	4.63	0.95	0.41	−2.01

**Table 2:** Band gap, effective mass and exciton binding energy values of Rb_2_InCuX_6_ (X = F, Cl, Br) calculated using the modified Becke–Johnson (TB-mBJ) potential and including spin–orbit coupling (mBJ + SOC).

Compounds	TB-mBJ (eV)	mBJ + SOC (eV)	m_h_*/m_o_	m_e_*/m_o_	$E_{b}^{ex}$
**Rb_2_InCuF_6_**	1.54	1.49	3.92	0.06	0.41
**Rb_2_InCuCl_6_**	0.94	0.91	0.48	0.03	0.16
**Rb_2_InCuBr_6_**	0.58	0.56	0.40	0.02	0.03

## Data Availability

The original contributions presented in this study are included in the article/Supplementary Materials. Further inquiries can be directed to the corresponding authors.

## Supplementary Materials

The following supporting information can be downloaded at: https://www.mdpi.com/article/10.3390/nano16100610/s1, Figure S1. Phase stability diagram of Rb_2_InCuX_6_ (X = F, Cl, Br) based on tolerance factor and formation investigated lie among the stable region, confirming the framework stability.
