# Supplementary material for: First-Principles Insights into the Structural, Electronic, Optical, and Thermoelectric Properties of Novel Halide Double Perovskites Rb2InCuX6 (X = F, Cl, Br)
Source: Nanomaterials (Basel). 2026 May 16;16(10):610. doi: 10.3390/nano16100610 (PMC13210047; doi:10.3390/nano16100610)
Supplement: Supplementary file 1 [file nanomaterials-16-00610-s001.zip › nanomaterials-4281187-supplementary.pdf]

Supporting Information

First-Principles Insights into the Structural, Electronic, Optical, and Thermoelectric Properties of Novel Halide Double Perovskites Rb_2InCuX_6 (X = F, Cl, Br)

Nabeel Israr [1], Peichao Zhu [1], Fawad Ali [1], Zubair Maroof [1], Shuaiqi He [1], Puyang Wu [1], Haoyang Lu [1], Weijia Sun [1], Zhaoxin Wu [1,2,*] and Fang Yuan [1,*]

[1] Key Laboratory for Physical Electronics and Devices of the Ministry of Education & Shaanxi Key Lab of Information Photonic Technique, School of Electronic Science and Engineering, Xi'an Jiaotong University, Xi'an 710049, China

[2] Collaborative Innovation Center of Extreme Optics, Shanxi University, Taiyuan 030006, China

* Corresponding Authors:

E-mail addresses: zhaoxinwu@mail.xjtu.edu.cn (Z. Wu)

yuanf121@xjtu.edu.cn (F. Yuan)

1. Supplementary Figure S1

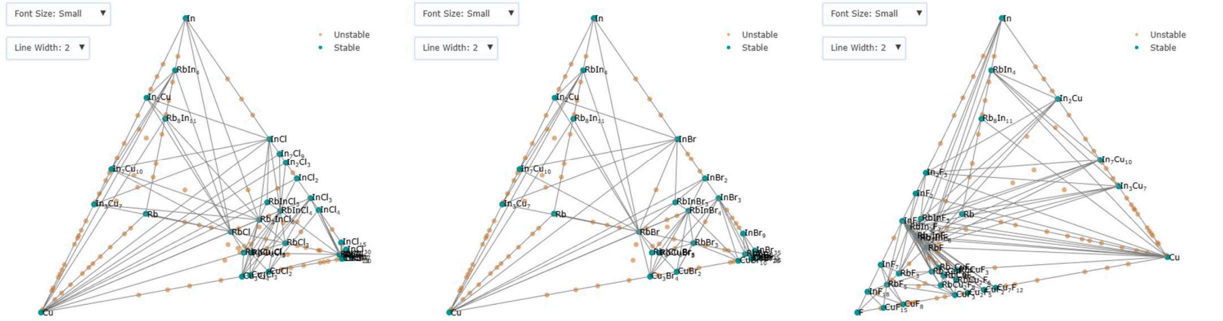

Figure S1. Phase stability diagram of Rb_2InCuX_6 (X = F, Cl, Br) based on tolerance factor and formation investigated lie among the stable region, confirming the framework stability.
